# Supplementary material for: How and why plants and human N-glycans are different: Insight from molecular dynamics into the “glycoblocks” architecture of complex carbohydrates
Source: Beilstein J Org Chem. 2020 Aug 21;16:2046–56. doi: 10.3762/bjoc.16.171 (PMC7445399; doi:10.3762/bjoc.16.171)
Supplement: File 1 — Computational methods and supplementary figures and tables. [file Beilstein_J_Org_Chem-16-2046-s001.pdf]

BEILSTEIN JOURNAL OF ORGANIC CHEMISTRY

Supporting Information

for

How and why plants and human N-glycans are different: Insight from molecular dynamics into the “glycoblocks” architecture of complex carbohydrates

Carl A. Fogarty, Aoife M. Harbison, Amy R. Dugdale and Elisa Fadda

Beilstein J. Org. Chem. **2020,** *16,* 2046–2056. doi:10.3762/bjoc.16.171

Computational methods and supplementary figures and tables

License and Terms: This is a supporting information file under the terms of the Creative Commons Attribution License (https://creativecommons.org/licenses/by/4.0). Please note that the reuse, redistribution and reproduction in particular requires that the authors and source are credited.
The license is subject to the *Beilstein Journal of Organic Chemistry* terms and conditions: (https://www.beilstein-journals.org/bjoc)

1. Computational Methods

System preparation. All N-glycan starting structures for the MD simulations were generated with the GLYCAM Carbohydrate Builder (http://www.glycam.org). For each sequence we selected the complete set of rotamers obtained by variation of the 1-6 torsion angles, namely *gg*, *gt* and *tg* conformations for each 1-6 torsion. The topology file corresponding to each structure was obtained using *tleap*[1], with parameters from the GLYCAM06-j1[2] for the carbohydrate atoms and with TIP3P for water molecules[3]. Each N-glycan was placed in the centre of a cubic simulation box of 16 Å sides with no counterions to be consistent with the simulations run in earlier work[4]. Long range electrostatic were treated by Particle Mesh Ewald (PME) with cut-off set at 11 Å and a B-spline interpolation for mapping particles to and from the mesh of order of 4. Van der Waals (vdW) interactions were cut-off at 11 Å. The MD trajectories were generated by Langevin dynamics[5,6] with collision frequency of 1.0 ps^{-1}. Pressure was kept constant by isotropic pressure scaling[7] with a pressure relaxation time of 2.0 ps. Integration was done with a time step of 0.002 ps for all simulations, with bonds to hydrogen atoms restrained with the SHAKE algorithm[8]. All calculations were run with the AMBER18 software package[1] on NVIDIA Tesla V100 16GB PCIe (Volta architecture) GPUs installed on the HPC infrastructure *kay* at the Irish Centre for High-End Computing (ICHEC).

Simulation protocol. The energy of the hydrated systems was initially minimized through 500,000 cycles of steepest descent, with all heavy atoms restrained with a harmonic potential with a force constant of 5 kcal $mol^{-1}Å^{-2}$. After minimization, the system was heated in two stages. During the first stage the temperature was raised from 0 to 100 K over 500 ps at constant volume and in the second stage from 100 K to 300 K over 500 ps at constant pressure. Through the heating process all heavy atoms were kept restrained. After heating phase all restraints were removed and the system was allowed to equilibrate for 5 ns at 300 K and at 1 atm of pressure. Separate production steps of 500 ns each were run for each rotamer (starting system) and convergence was assessed based on conformational and clustering analysis. Simulations were extended, if the sampling was not deemed as fully converged.

Data analysis. All trajectories were processed using *cpptraj*[1] and visually analysed with the Visual Molecular Dynamics (VMD) software package[9]. Backbone Root Mean Square Deviation (RMSD) and torsion angles values were measured using VMD. A density-based clustering method was used to calculate the populations of occupied conformations for each torsion angle in a trajectory and heat maps for each dihedral were generated with a kernel density estimate (KDE) function. Statistical and clustering analysis was done with the R package and data were plotted with RStudio (www.rstudio.com).

2. Supplementary tables and figures

Table S1. Results of the clustering analysis showing the median and standard deviation values (in parenthesis) for the torsion angles (°) measured through a cumulative 1.5 μs MD sampling of the α(1-3) core fucosylated *ngf* glycan.

**Fuc α(1-3) GlcNAc**	**Phi**	**Psi**	**Pop(%)**
Cluster 1	-71.1 (8.9)	141.1 (6.3)	100
**GlcNAc β(1-4) GlcNAc**	**Phi**	**Psi**	**Pop(%)**
Cluster 1	-72.1 (8.3)	-107.1 (7.6)	100
**Man β(1-4) GlcNAc**	**Phi**	**Psi**	**Pop(%)**
Cluster 1	-73.3 (12.3)	-122.1 (14.9)	90.0
Cluster 2	-166.4 (16.7)	-145.6 (9.8)	8.3
Cluster 3	-64.0 (10.0)	75.3 (9.3)	1.7
**Man α(1-6) Man**	**Phi**	**Psi**	**Pop(%)**
Cluster 1	69.2 (10.2)	-176.6 (17.5)	65.7
Cluster 2	74.2 (9.4)	89.2 (10.8)	25.2
Cluster 3	60.4 (6.4)	60.0 (5.8)	9.1
**GlcNAc β(1-2) Man (*1-6*)**	**Phi**	**Psi**	**Pop(%)**
Cluster 1	-78.8 (14.3)	160.7 (20.6)	96.2
Cluster 2	69.6 (9.3)	154.8 (10.3)	2.5
**Man α(1-3) Man**	**Phi**	**Psi**	**Pop(%)**
Cluster 1	70.9 (8.5)	140.3 (13.5)	66.8
Cluster 2	70.6 (8.7)	99.2 (9.5)	33.2
**GlcNAc β(1-2) Man (*1-3*)**	**Phi**	**Psi**	**Pop(%)**
Cluster 1	-77.8 (13.7)	161.5 (13.5)	88.8
Cluster 2	-78.5 (8.0)	109.2 (7.2)	9.7

Table S2. Results of the clustering analysis showing the median and standard deviation values (in parenthesis) for the torsion angles (°) measured through a cumulative 1.5 μs MD sampling of the α(1-3) core fucosylated *gf* glycan.

**Fuc α(1-3) GlcNAc**	**Phi**	**Psi**	**Pop(%)**
Cluster 1	-72.5 (10.7)	125.2 (17.4)	100
**GlcNAc β(1-4) GlcNAc**	**Phi**	**Psi**	**Pop(%)**
Cluster 1	-72.9 (8.6)	-105.7 (11.7)	100
**Man β(1-4) GlcNAc**	**Phi**	**Psi**	**Pop(%)**
Cluster 1	-74.0 (12.3)	-123.9 (15.7)	88.5
Cluster 2	-165.7 (14.7)	-145.7 (8.8)	11.5
**Man α(1-6) Man**	**Phi**	**Psi**	**Pop(%)**
Cluster 1	69.3 (10.6)	-177.6 (19.4)	72.5
Cluster 2	71.5 (6.2)	79.2 (7.1)	16.2
Cluster 3	60.7 (5.7)	60.4 (5.1)	1.1
**GlcNAc β(1-2) Man (*1-6*)**	**Phi**	**Psi**	**Pop(%)**
Cluster 1	-76.7 (13.8)	162.5 (12.4)	88.4
Cluster 2	-76.0 (6.2)	113.5 (6.6)	5.9
Cluster 3	-147.6 (9.5)	97.9 (9.4)	1.9
**Gal β(1-3) GlcNAc (*1-6*)**	**Phi**	**Psi**	**Pop(%)**
Cluster 1	-72.9 (11.2)	124.0 (18.2)	96.6
Cluster 2	-82.1 (12.2)	-64.0 (8.4)	3.0
Cluster 3	-150.0 (11.3)	-101.0 (7.3)	1.4
**Man α(1-3) Man**	**Phi**	**Psi**	**Pop(%)**
Cluster 1	71.7 (8.9)	141.5 (14.5)	66.9
Cluster 2	70.4 (8.6)	99.12 (9.5)	33.1
**GlcNAc β(1-2) Man (*1-3*)**	**Phi**	**Psi**	**Pop(%)**
Cluster 1	-77.9 (14.2)	160.9 (14.5)	83.3
Cluster 2	67.2 (9.2)	152.4 (10.4)	8.6
Cluster 3	-77.7 (7.7)	152.4 (6.2)	8.1
**Gal β(1-3) GlcNAc (*1-3*)**	**Phi**	**Psi**	**Pop(%)**
Cluster 1	-72.4 (9.8)	125.0 (15.8)	100

Table S3. Results of the clustering analysis showing the median and standard deviation values (in parenthesis) for the torsion angles (°) measured through a cumulative 3 μs MD sampling of the α(1-2) core xylosylated *ngx* glycan.

**GlcNAc β(1-4) GlcNAc**	**Phi**	**Psi**	**Pop(%)**
Cluster 1	-78.1 (10.2)	-129.6 (15.67)	96.3
Cluster 2	-81.8 (9.8)	63.8 (8.11)	3.7
**Man β(1-4) GlcNAc**	**Phi**	**Psi**	**Pop(%)**
Cluster 1	-75.5 (12.6)	-123.5 (14.6)	94.4

Cluster 2	-64.2 (6.8)	74.2 (9.7)	5.6
**Xyl β(1-2) Man**	**Phi**	**Psi**	**Pop(%)**
Cluster 1	-80.2 (12.0)	133.2 (17.0)	100
**Man α(1-6) Man**	**Phi**	**Psi**	**Pop(%)**
Cluster 1	69.7 (10.3)	-174.4 (16.8)	77.2
Cluster 2	73.5 (10.1)	106.1 (10.6)	22.8
**GlcNAc β(1-2) Man (*1-6*)**	**Phi**	**Psi**	**Pop(%)**
Cluster 1	-80.9 (15.5)	161.7 (12.0)	90.1
Cluster 2	-73.1 (7.9)	114.2 (7.8)	9.3
**Man α(1-3) Man**	**Phi**	**Psi**	**Pop(%)**
Cluster 1	78.4 (7.3)	114.8 (14.8)	100
**GlcNAc β(1-2) Man (*1-3*)**	**Phi**	**Psi**	**Pop(%)**
Cluster 1	-77.4 (13.3)	161.5 (12.2)	88.2
Cluster 2	-78.9 (6.4)	109.1 (7.18)	8.2
Cluster 3	-66.9 (9.0)	149.1 (12.3)	3.6

Table S4. Results of the clustering analysis showing the median and standard deviation values (in parenthesis) for the torsion angles (°) measured through a cumulative 1.5 μs MD sampling of the α(1-3) core xylosylated *gx* glycan.

**GlcNAc β(1-4) GlcNAc**	**Phi**	**Psi**	**Pop(%)**
Cluster 1	-78.0 (10.1)	-130.2(15.7)	97.5
Cluster 2	-84.0 (5.4)	-64.8 (5.6)	2.5
**Man β(1-4) GlcNAc**	**Phi**	**Psi**	**Pop(%)**
Cluster 1	-75.2 (13.5)	-124.7 (15.0)	87.6
Cluster 2	-67.9 (14.7)	72.9 (11.7)	11.3
Cluster 3	-178.3 (6.9)	-175.5 (7.3)	1.0
**Xyl β(1-2) Man**	**Phi**	**Psi**	**Pop(%)**
Cluster 1	-78.2 (9.3)	138.4 (15.4)	100
**Man α(1-6) Man**	**Phi**	**Psi**	**Pop(%)**
Cluster 1	70.5 (10.4)	-173.6 (19.4)	70.0
Cluster 2	71.8 (9.6)	103.8 (12.9)	25.7
Cluster 3	161.6 (8.0)	132.5 (9.2)	2.3
Cluster 4	79.0 (6.7)	-80.2 (8.05)	1.59
**GlcNAc β(1-2) Man (*1-6*)**	**Phi**	**Psi**	**Pop(%)**
Cluster 1	-82.1 (15.0)	161.3 (12.2)	88.2
Cluster 2	-77.7 (7.47)	112.5 (7.0)	10.0
Cluster 3	-147.3 (9.0)	98.9 (9.4)	1.9
**Gal β(1-3) GlcNAc (*1-6*)**	**Phi**	**Psi**	**Pop(%)**

Cluster 1	-72.1 (10.0)	126.1 (15.5)	80.8
Cluster 2	-83.6 (11.4)	-63.0 (7.57)	19.2
**Man α(1-3) Man**	**Phi**	**Psi**	**Pop(%)**
Cluster 1	72.5 (10.7)	125.2 (17.4)	100
**GlcNAc β(1-2) Man (*1-3*)**	**Phi**	**Psi**	**Pop(%)**
Cluster 1	-78.5 (14.2)	162.4 (13.0)	91.2
Cluster 2	-77.8 (7.4)	111.1 (7.0)	8.8
**Gal β(1-3) GlcNAc (*1-3*)**	**Phi**	**Psi**	**Pop(%)**
Cluster 1	-71.1 (7.2)	125.5 (11.8)	100

Table S5. Results of the clustering analysis showing the median and standard deviation values (in parenthesis) for the torsion angles (°) measured through a cumulative 1.5 μs MD sampling of the β(1-2) core xylosylated and α(1-3) core fucosylated *ngxf* glycan.

**Fuc α(1-3) GlcNAc**	**Phi**	**Psi**	**Pop(%)**
Cluster 1	-71.3 (9.0)	140.5 (6.0)	100
**GlcNAc β(1-4) GlcNAc**	**Phi**	**Psi**	**Pop(%)**
Cluster 1	-73.2 (11.0)	-106.4 (21.0)	100
**Man β(1-4) GlcNAc**	**Phi**	**Psi**	**Pop(%)**
Cluster 1	-75.9 (14.4)	-124.2 (15.3)	75.4
Cluster 2	-66.5 (11.3)	73.3 (10.8)	21.1
Cluster 3	179.0 (7.6)	-174.7 (8.2)	2.3
Cluster 4	-151.9 (8.2)	-146.5 (6.7)	1.2
**Xyl β(1-2) Man**	**Phi**	**Psi**	**Pop(%)**
Cluster 1	-78.8 (10.8)	135.9 (17.4)	100
**Man α(1-6) Man**	**Phi**	**Psi**	**Pop(%)**
Cluster 1	70.6 (9.0)	175.2 (17.6)	68.5
Cluster 2	71.7 (10.0)	106.5 (12.3)	24.5
Cluster 3	81.6 (8.0)	-75.0 (10.0)	4.4
Cluster 4	158.0 (8.9)	135.9 (10.4)	2.4
**GlcNAc β(1-2) Man (*1-6*)**	**Phi**	**Psi**	**Pop(%)**
Cluster 1	-79.2 (14.4)	158.4 (24.9)	90.1
Cluster 2	-80.0 (6.33)	113.0 (7.3)	9.3
**Man α(1-3) Man**	**Phi**	**Psi**	**Pop(%)**
Cluster 1	69.1 (9.4)	113.8 (16.8)	1
**GlcNAc β(1-2) Man (*1-3*)**	**Phi**	**Psi**	**Pop(%)**
Cluster 1	-77.4 (13.3)	161.5 (12.2)	88.2
Cluster 2	-78.9 (6.4)	109.1 (7.18)	8.21
Cluster 3	-66.9 (9.0)	149.1 (12.3)	3.58

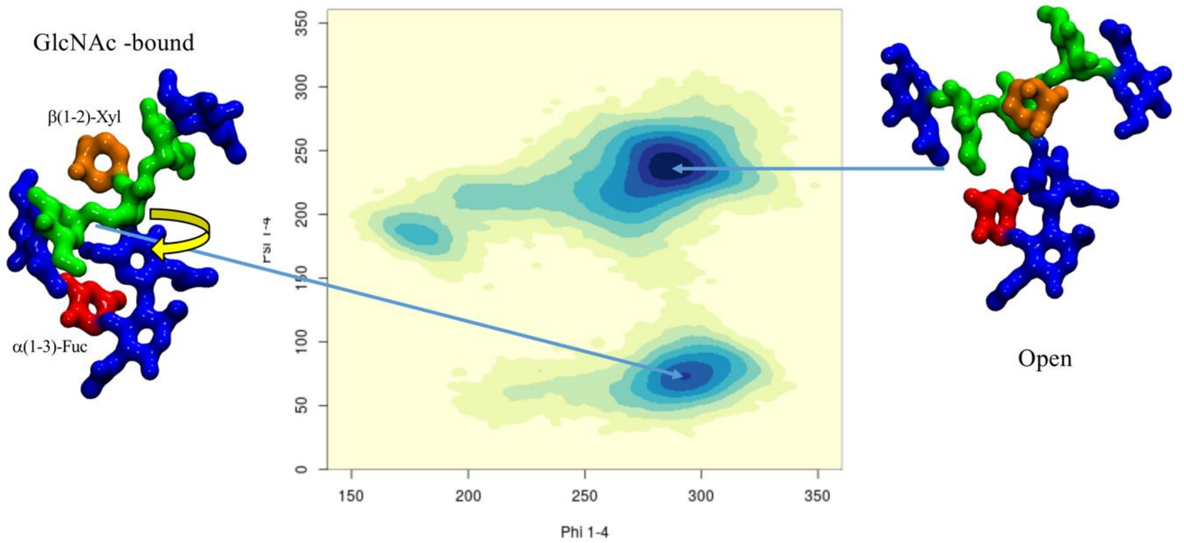

Figure S1. Conformational analysis of the *ngxf* glycoform in terms of phi/psi torsion values, shown on the x and y axis, respectively, of the core Man-β(1-4)-GlcNAc linkage. Representative structures selected from MD sampling are shown on the left- and right-hand side of the heat map. The GlcNAc-bound conformation is obtained through a torsion of the Man3 "glycoblock" relative to the chitobiose and it is stabilized by hydrogen bonding interactions between the β(1-2)-Xyl and α(1-3)-Fuc. The monosaccharides colouring follows the SFNG nomenclature. The structure rendering was done with VMD and the graphical statistical analysis with *RStudio* (www.rstudio.com).

Table S6 Results of the clustering analysis showing the median and standard deviation values (in parenthesis) for the torsion angles (°) measured through a cumulative 1.5 μs MD sampling of the β(1-2) xylosylated and α(1-3) core fucosylated *gxf* glycan

**Fuc α(1-3) GlcNAc**	**Phi**	**Psi**	**Pop(%)**
Cluster 1	-71.1 (9.8)	140.5 (7.1)	88.9
Cluster 2	-156.8 (5.6)	90.7 (6.7)	11.1
**GlcNAc β(1-4) GlcNAc**	**Phi**	**Psi**	**Pop(%)**
Cluster 1	-72.2 (8.5)	-107.0 (8.0)	100
**Man β(1-4) GlcNAc**	**Phi**	**Psi**	**Pop(%)**
Cluster 1	-76.7 (15.1)	-124.5 (17.0)	85.2
Cluster 2	-179.5 (8.5)	-174.7 (9.5)	8.3
Cluster 3	-63.0 (9.9)	-75.6 (10.3)	5.5
Cluster 4	-153.1 (6.2)	-147.4 (5.31)	1
**Xyl β(1-2) Man**	**Phi**	**Psi**	**Pop(%)**
Cluster 1	-78.2 (9.9)	135.9 (17.4)	100
**Man α(1-6) Man**	**Phi**	**Psi**	**Pop(%)**
Cluster 1	70.73 (10.3)	-174.2 (19.0)	87.2
Cluster 2	70.8 (8.1)	101.5 (10.7)	9.3
Cluster 3	161.6 (8.5)	131.9 (10.6)	3.5
**GlcNAc β(1-2) Man (*1-6*)**	**Phi**	**Psi**	**Pop(%)**

Cluster 1	-78.1 (14.3)	161.9 (12.9)	84.7
Cluster 2	-76.1 (7.6)	111.6 (7.2)	10.5
Cluster 3	-147.1 (10.9)	99.5 (10.1)	3.2
Cluster 4	67.4 (7.4)	152.4 (8.4)	1.6
**Gal β(1-3) GlcNAc (*1-6*)**	**Phi**	**Psi**	**Pop(%)**
Cluster 1	-70.7 (10.4)	125.0 (16.8)	96.7
Cluster 2	-81.6 (7.07)	-64.6 (6.4)	3.3
Cluster 3	-81.6 (7.07)	-64.6 (6.4)	1.3
**Man α(1-3) Man**	**Phi**	**Psi**	**Pop(%)**
Cluster 1	68.7 (9.1)	114.1 (16.6)	1
**GlcNAc β(1-2) Man (*1-3*)**	**Phi**	**Psi**	**Pop(%)**
Cluster 1	-78.2 (14.7)	162.0 (13.0)	90.1
Cluster 2	-78.1 (7.2)	112.1 (6.8)	9.9
**Gal β(1-3) GlcNAc (*1-3*)**	**Phi**	**Psi**	**Pop(%)**
Cluster 1	-71.9 (6.9)	126.0 (11.9)	100

Table S7. Results of the clustering analysis showing the median and standard deviation values (in parenthesis) for the torsion angles (°) measured through a cumulative 1.5 µs MD sampling of the α(1-3) core fucosylated LeA glycan.

**Fuc α(1-3) GlcNAc**	**sPhi**	**Psi**	**Pop(%)**
Cluster 1	-71.7 (9.8)	141.8 (6.6)	82.8
Cluster 2	-156.3 (7.02)	88.8 (17.6)	17.2
**GlcNAc β(1-4) GlcNAc**	**Phi**	**Psi**	**Pop(%)**
Cluster 1	-72.6 (8.7)	-107.4 (7.4)	100
**Man β(1-4) GlcNAc**	**Phi**	**Psi**	**Pop(%)**
Cluster 1	-72.2 (12.0)	-124.5 (14.0)	97.1
Cluster 2	179.7 (6.1)	-178.2 (6.6)	2.9
**Man α(1-6) Man**	**Phi**	**Psi**	**Pop(%)**
Cluster 1	-75.5 (7.4)	148.1 (7.4)	53.6
Cluster 2	-70.1 (5.8)	-177.0 (11.2)	28.2
Cluster 3	-73.2 (6.7)	-101.9 (6.0)	9.4
Cluster 4	-148.0 (6.3)	-165.3 (5.8)	8.8
**Xyl β(1-2) Man**	**Phi**	**Psi**	**Pop(%)**
Cluster 1	-83.9 (10.5)	132.8 (14.0)	100
**GlcNAc β(1-2) Man (*1-6*)**	**Phi**	**Psi**	**Pop(%)**
Cluster 1	-150.0 (9.3)	98.5 (7.9)	40.9
Cluster 2	-91.5 (8.5)	151.6 (8.6)	29.9
Cluster 3	-62.7 (8.5)	161.4 (9.9)	29.2
**Gal β(1-3) GlcNAc (*1-6*)**	**Phi**	**Psi**	**Pop(%)**

Cluster 1	-70.1 (7.6)	131.5 (7.6)	100
**Fuc α(1-4) GlcNAc (*1-6*)**	**Phi**	**Psi**	**Pop(%)**
Cluster 1	-67.8 (7.5)	-101.26 (7.3)	100
**Man α(1-3) Man**	**Phi**	**Psi**	**Pop(%)**
Cluster 1	61.5 (8.5)	111.0 (15.1)	100
**GlcNAc β(1-2) Man (*1-3*)**	**Phi**	**Psi**	**Pop(%)**
Cluster 1	-77.9 (16.9)	162.3 (12.3)	57.8
Cluster 2	-79.9 (11.4)	105.1 (12.4)	27.9
Cluster 3	66.1 (10.1)	153.8 (10.8)	14.26
**Gal β(1-3) GlcNAc (*1-3*)**	**Phi**	**Psi**	**Pop(%)**
Cluster 1	-70.6 (7.5)	134.5 (6.8)	100
**Fuc α(1-4) GlcNAc (*1-3*)**	**Phi**	**Psi**	**Pop(%)**
Cluster 1	-68.6 (8.9)	-100.9 (6.7)	98.4
Cluster 2	-148.9 (8.0)	-150.7 (3.8)	1.6

Table S8. Results of the clustering analysis showing the median and standard deviation values (in parenthesis) for the torsion angles (°) measured through a cumulative 1.5 µs MD sampling of the β(1-2) xylosylated *mgx* glycan. Note: *mg* refers to the mammalian terminal β(1-4)-Gal.

**GlcNAc β(1-4) GlcNAc**	**Phi**	**Psi**	**Pop(%)**
Cluster 1	-78.2 (10.9)	-131.1 (15.8)	97.5
Cluster 2	-79.6 (11.3)	66.6 (11.5)	2.5
**Man β(1-4) GlcNAc**	**Phi**	**Psi**	**Pop(%)**
Cluster 1	-75.7 (17.1)	-123.7 (14.7)	91.3
Cluster 2	-68.1 (12.6)	72.1 (11.9)	8.7
**Xyl β(1-2) Man**	**Phi**	**Psi**	**Pop(%)**
Cluster 1	-81.7 (19.1)	133.6 (20.1)	100
**Man α(1-6) Man**	**Phi**	**Psi**	**Pop(%)**
Cluster 1	72.2 (9.4)	103.5 (11.3)	56.9
Cluster 2	69.9 (8.3)	-173.8 (15.7)	39.8
Cluster 3	162.2 (9.1)	131.2 (9.3)	1.7
Cluster 4	58.5 (3.2)	59.98 (4.3)	1.6
**GlcNAc β(1-2) Man (*1-6*)**	**Phi**	**Psi**	**Pop(%)**
Cluster 1	-78.1 (14.3)	161.9 (12.9)	84.7
Cluster 2	-76.1 (7.6)	111.6 (7.2)	10.5
Cluster 3	-147.1 (10.9)	99.5 (10.1)	3.2
Cluster 4	67.4 (7.4)	152.4 (8.4)	1.6
**Gal β(1-4) GlcNAc (*1-6*)**	**Phi**	**Psi**	**Pop(%)**
Cluster 1	-72.9 (15.8)	-119.9 (16.0)	98.9

Cluster 2	-73.5 (12.6)	-73.24 (12.5)	0.6
Cluster 3	63.6 (10.7)	-117.8 (6.8)	0.8
**Man α(1-3) Man**	**Phi**	**Psi**	**Pop(%)**
Cluster 1	68.9 (9.7)	114.5 (16.7)	1
**GlcNAc β(1-2) Man (*1-3*)**	**Phi**	**Psi**	**Pop(%)**
Cluster 1	-79.0 (13.8)	162.5 (12.2)	87.5
Cluster 2	-80.9 (9.12)	109.4 (9.0)	12.5
**Gal β(1-4) GlcNAc (*1-3*)**	**Phi**	**Psi**	**Pop(%)**
Cluster 1	-72.41 (10.9)	-118.4 (15.1)	100

Table S9. Results of the clustering analysis showing the median and standard deviation values (in parenthesis) for the torsion angles (°) measured through a cumulative 4.5 μs MD sampling of the β(1-2) xylosylated and α(1-6) core fucosylated *mgmfx* glycan. Note: *mg* refers to the mammalian terminal β(1-4)-Gal and *mf* to the mammalian core α(1-6)-Fuc.

**Fuc α(1-6) GlcNAc**	**Phi**	**Psi**	**Pop(%)**
Cluster 1	-74.5 (9.6)	172.4 (14.5)	92.1
Cluster 2	-95.9 (4.5)	71.78 (6.1)	6.9
Cluster 3	-75.6 (2.5)	1113.8 (2.17)	1.0
**GlcNAc β(1-4) GlcNAc**	**Phi**	**Psi**	**Pop(%)**
Cluster 1	-77.2 (9.5)	-126.0 (14.3)	100
**Man β(1-4) GlcNAc**	**Phi**	**Psi**	**Pop(%)**
Cluster 1	-74.3 (15.0)	-122.7 (14.2)	97.1
Cluster 2	-67.4 (10.1)	73.6 (10.7)	2.9
**Man α(1-6) Man**	**Phi**	**Psi**	**Pop(%)**
Cluster 1	71.8 (9.4)	103.3 (10.98)	70.0
Cluster 2	70.4 (9.0)	-177.0 (11.2)	26.9
Cluster 3	67.5 (5.24)	-62.5 (5.7)	3.1
**Xyl β(1-2) Man**	**Phi**	**Psi**	**Pop(%)**
Cluster 1	-83.9 (10.5)	132.8 (14.0)	100
**GlcNAc β(1-2) Man (*1-6*)**	**Phi**	**Psi**	**Pop(%)**
Cluster 1	-91.5 (12.5)	159.9 (10.3)	96.3
Cluster 2	-75.5 (3.9)	113.6 (3.8)	2.9
Cluster 3	66.0 (6.8)	154.3 (5.8)	0.8
**Gal β(1-4) GlcNAc (*1-6*)**	**Phi**	**Psi**	**Pop(%)**
Cluster 1	-74.9 (13.56)	-122.1 (15.5)	97.5
Cluster 2	-83.6 (18.6)	65.3 (13.5)	2.5
**Man α(1-3) Man**	**Phi**	**Psi**	**Pop(%)**
Cluster 1	68.7 (9.9)	115.6 (16.9)	100
**GlcNAc β(1-2) Man (*1-3*)**	**Phi**	**Psi**	**Pop(%)**

Cluster 1	-79.1 (13.2)	162.3 (11.4)	90.9
Cluster 2	-77.9 (6.8)	110.4 (7.6)	9.1
**Gal β(1-4) GlcNAc (*1-3*)**	**Phi**	**Psi**	**Pop(%)**
Cluster 1	-72.7 (15.4)	-118.6 (15.7)	97.3
Cluster 2	-74.0 (12.4)	70.12 (13.1)	2.7

Table S10. Results of the clustering analysis showing the median and standard deviation values (in parenthesis) for the torsion angles (°) measured through a cumulative 4.5 µs MD sampling of the β(1-2) xylosylated and α(1-3) core fucosylated *nmgmfx* glycan. Note: *nmg* refers to the absence of mammalian terminal β(1-4)-Gal and *mf* to the mammalian core α(1-6)-Fuc.

**Fuc α(1-6) GlcNAc**	**Phi**	**Psi**	**Pop(%)**
Cluster 1	-73.0 (9.6)	177.0 (14.8)	93.1
Cluster 2	-95.6 (4.0)	75.0 (5.4)	6.9
**GlcNAc β(1-4) GlcNAc**	**Phi**	**Psi**	**Pop(%)**
Cluster 1	-77.2 3(9.7)	-126.0 (14.4)	100
**Man β(1-4) GlcNAc**	**Phi**	**Psi**	**Pop(%)**
Cluster 1	-76.6 (18.5)	-124.8 (16.9)	91.1
Cluster 2	-66.2 (12.5)	73.0 (11.9)	8.2
**Man α(1-6) Man**	**Phi**	**Psi**	**Pop(%)**
Cluster 1	70.1 (9.2)	-173.6 (14.6)	71.5
Cluster 2	72.1 (8.3)	104.25 (9.7)	28.5
**Xyl β(1-2) Man**	**Phi**	**Psi**	**Pop(%)**
Cluster 1	-80.6 (16.5)	135.8 (14.0)	100
**GlcNAc β(1-2) Man (*1-6*)**	**Phi**	**Psi**	**Pop(%)**
Cluster 1	-82.8 (14.94)	161.1 (11.9)	90.6
Cluster 2	-77.8 (7.3)	111.3 (6.8)	7.4
Cluster 3	66.0 (7.3)	152.9 (8.7)	2.0
**Man α(1-3) Man**	**Phi**	**Psi**	**Pop(%)**
Cluster 1	68.9 (9.4)	114.4 (16.9)	100
**GlcNAc β(1-2) Man (*1-3*)**	**Phi**	**Psi**	**Pop(%)**
Cluster 1	-78.4 (13.4)	162.6 (11.4)	88.8
Cluster 2	-78.9 (7.2)	110.4 (7.0)	9.0
Cluster 2	66.45 (10.9)	152.6 (10.7)	2.2

Table S11. Results of the clustering analysis showing the median and standard deviation values (in parenthesis) for the torsion angles (°) measured through a cumulative 1.5 μs MD sampling of the β(1-2) xylosylated and α(1-3) core fucosylated *mgpfx* glycan. Note: *mg* refers to the mammalian terminal β(1-4)-Gal and *pf* to the plant core α(1-3)-Fuc.

**Fuc α(1-3) GlcNAc**	**Phi**	**Psi**	**Pop(%)**
Cluster 1	-70.8 (10.7)	141.3 (8.9)	93.8
Cluster 2	-156.8 (7.7)	91.3 (9.3)	6.2
**GlcNAc β(1-4) GlcNAc**	**Phi**	**Psi**	**Pop(%)**
Cluster 1	-72.5 (8.8)	-107.3 (9.1)	100
**Man β(1-4) GlcNAc**	**Phi**	**Psi**	**Pop(%)**
Cluster 1	-76.4 (15.1)	-123.6 (17.3)	87.0
Cluster 2	-68.2 (12.8)	70.9 (11.2)	13.0
**Xyl β(1-2) Man**	**Phi**	**Psi**	**Pop(%)**
Cluster 1	-80.7 (15.3)	134.5 (16.5)	100
**Man α(1-6) Man**	**Phi**	**Psi**	**Pop(%)**
Cluster 1	69.9 (9.0)	-175.5 (15.0)	49.1
Cluster 2	72.4 (9.7)	105.1 (12.5)	46.7
Cluster 3	158.6 (11.8)	135.8 (13.6)	4.2
**GlcNAc β(1-2) Man (*1-6*)**	**Phi**	**Psi**	**Pop(%)**
Cluster 1	-86.2 (15.0)	160.9 (11.4)	96.4
Cluster 2	-78.5 (6.1)	113.5 (4.9)	3.6
**Gal β(1-4) GlcNAc (*1-6*)**	**Phi**	**Psi**	**Pop(%)**
Cluster 1	-72.9 (15.0)	-119.9 (15.6)	97.2
Cluster 2	-74.1 (13.1)	70.1 (13.8)	2.8
**Man α(1-3) Man**	**Phi**	**Psi**	**Pop(%)**
Cluster 1	68.8 (9.6)	114.5 (16.7)	1
**GlcNAc β(1-2) Man (*1-3*)**	**Phi**	**Psi**	**Pop(%)**
Cluster 1	-78.8 (13.8)	162.6 (12.6)	88.3
Cluster 2	-80.7 (8.0)	109.5 (7.6)	9.6
Cluster 3	65.2 (9.9)	150.8 (10.8)	2.1
**Gal β(1-4) GlcNAc (*1-3*)**	**Phi**	**Psi**	**Pop(%)**
Cluster 1	-72.5 (11.5)	-118.6 (15.5)	100

Table S12 Results of the clustering analysis showing the median and standard deviation values (in parenthesis) for the torsion angles (°) measured through a cumulative 1.5 μs MD sampling of the α(1-3) core fucosylated *mgpf* glycan. Note: *mg* refers to the mammalian terminal β(1-4)-Gal and *pf* to the plant core α(1-3)-Fuc.

**Fuc α(1-3) GlcNAc**	**Phi**	**Psi**	**Pop(%)**
Cluster 1	-71.1 (10.6)	140.3 (14.8)	98.1
Cluster 2	-156.5 (6.2)	91.4 (9.3)	1.9
**GlcNAc β(1-4) GlcNAc**	**Phi**	**Psi**	**Pop(%)**

Cluster 1	-73.0 (14.8)	-121.5 (15.7)	95.9
Cluster 2	-80.9 (15.3)	62.5 (13.4)	4.1
**Man β(1-4) GlcNAc**	**Phi**	**Psi**	**Pop(%)**
Cluster 1	-76.6 (14.1)	-124.9 (16.1)	77.9
Cluster 2	-153.7 (13.3)	-139.7 (8.5)	12.8
Cluster 3	-71.1 (12.5)	69.6 (11.4)	9.3
**Man α(1-6) Man**	**Phi**	**Psi**	**Pop(%)**
Cluster 1	74.2 (13.0)	86.5 (14.7)	74.8
Cluster 2	70.3 (9.1)	-176.5 (14.7)	25.2
**GlcNAc β(1-2) Man (*1-6*)**	**Phi**	**Psi**	**Pop(%)**
Cluster 1	-80.2 (14.6)	163.2 (12.4)	100
**Gal β(1-4) GlcNAc (*1-6*)**	**Phi**	**Psi**	**Pop(%)**
Cluster 1	-73.0 (14.88)	-121.58 (15.8)	95.9
Cluster 2	-80.9 (15.3)	62.5 (13.4)	4.1
**Man α(1-3) Man**	**Phi**	**Psi**	**Pop(%)**
Cluster 1	70.95 (9.6)	140.95 (15.2)	73.0
Cluster 1	70.1(8.21)	101.2 (8.8)	27.0
**GlcNAc β(1-2) Man (*1-3*)**	**Phi**	**Psi**	**Pop(%)**
Cluster 1	-78.5 (13.3)	162.1 (11.7)	91.2
Cluster 2	-77.9 (6.4)	110.7 (6.62)	8.8
**Gal β(1-4) GlcNAc (*1-3*)**	**Phi**	**Psi**	**Pop(%)**
Cluster 1	-72.2 (10.9)	-118.4 (15.0)	100

Table S13. Results of the clustering analysis showing the median and standard deviation values (in parenthesis) for the torsion angles (°) measured through a cumulative 2 μs MD sampling of the α(1-3) and α(1-6) core fucosylated *mgmfpf* glycan. Note: *mg* refers to the mammalian terminal β(1-4)-Gal, *pf* to the plant core α(1-3)-Fuc and *mf* to the mammalian core α(1-6)-Fuc.

**Fuc α(1-6) GlcNAc**	**Phi**	**Psi**	**Pop(%)**
Cluster 1	-72.0 (9.6)	-179.5 (14.8)	74.3
Cluster 2	-76.2 (4.0)	117.36 (12.1)	12.9
Cluster 3	-144.4 (7.7)	171.0 (5.5)	12.8
**Fuc α(1-3) GlcNAc**	**Phi**	**Psi**	**Pop(%)**
Cluster 1	-70.2 (10.3)	140.1 (9.7)	88.6
Cluster 2	-157.3 (7.7)	91.4 (8.9)	11.4
**GlcNAc β(1-4) GlcNAc**	**Phi**	**Psi**	**Pop(%)**
Cluster 1	-73.8 (9.5)	-106.4 (14.1)	91.7
Cluster 2	-154.9 (10.6)	-147.8 (7.4)	8.3
**Man β(1-4) GlcNAc**	**Phi**	**Psi**	**Pop(%)**
Cluster 1	-72.1 (10.9)	-120.7 (12.6)	74.8

Cluster 2	-153.0 (13.0)	-139.9 (8.3)	25.2
**Man α(1-6) Man**	**Phi**	**Psi**	**Pop(%)**
Cluster 1	73.5 (11.7)	86.6 (17.32)	85.1
Cluster 2	69.5 (9.9)	-176.3 (15.8)	14.9
**GlcNAc β(1-2) Man (*1-6*)**	**Phi**	**Psi**	**Pop(%)**
Cluster 1	-85.1 (14.9)	161.7 (13.4)	100
**Gal β(1-4) GlcNAc (*1-6*)**	**Phi**	**Psi**	**Pop(%)**
Cluster 1	-73.9 (11.5)	-123.5 (16.5)	98.7
Cluster 2	-146.2 (8.5)	-142.1 (6.9)	1.3
**Man α(1-3) Man**	**Phi**	**Psi**	**Pop(%)**
Cluster 1	71.1 (9.3)	140.5 (15.6)	72.9
Cluster 1	69.9 (8.8)	100.63(9.8)	27.1
**GlcNAc β(1-2) Man (*1-3*)**	**Phi**	**Psi**	**Pop(%)**
Cluster 1	-78.7 (13.8)	161.1 (12.8)	90.4
Cluster 2	-79.1 (7.7)	110.7 (7.2)	9.6
**Gal β(1-4) GlcNAc (*1-3*)**	**Phi**	**Psi**	**Pop(%)**
Cluster 1	-72.4 (10.9)	-118.6 (15.2)	95.0
Cluster 1	-143.2 (10.4)	-144.4 (5.9)	2.9
Cluster 1	-74.2 (9.3)	67.6 (9.4)	2.1

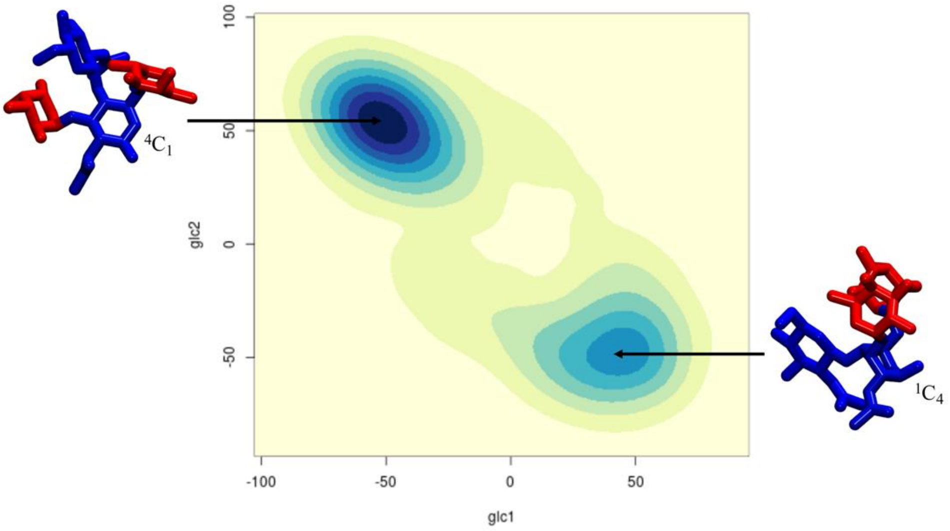

Figure S2. Conformational analysis N-linked GlcNAc pucker along the 1.5 μs cumulative sampling of the α(1-3) and α(1-6) core fucosylated A2G2 (*mgmfpf)* N-glycan. Representative structures of the fucosylated chitobiose selected from MD sampling of the whole N-glycans are shown on the left- and right-hand side of the heat map where the ring pucker is also indicated. The x and y axis are labelled with the torsion angled measured, namely C1C2C3C4 (glc1) and C2C3C4C5 (glc2), respectively. The monosaccharides colouring follows the SFNG nomenclature. The structure rendering was done with VMD and the graphical statistical analysis with *RStudio* (www.rstudio.com).

Table S14. Results of the clustering analysis showing the median and standard deviation values (in parenthesis) for the torsion angles (°) measured through a cumulative 2 μs MD sampling of the β(1-2) xylosylated and α(1-3) and α(1-6) core fucosylated *mgxmfpf* glycan. Note: *mg* refers to the mammalian terminal β(1-4)-Gal, *pf* to the plant core α(1-3)-Fuc and *mf* to the mammalian core α(1-6)-Fuc.

**Fuc α(1-6) GlcNAc**	**Phi**	**Psi**	**Pop(%)**
Cluster 1	-75.9 (11.25)	175.65 (14.0)	79.3
Cluster 2	-142.7 (9.8)	171.3(6.2)	16.9
Cluster 3	-95.48 (4.4)	71.95(4.8)	3.8
**Fuc α(1-3) GlcNAc**	**Phi**	**Psi**	**Pop(%)**
Cluster 1	-70.6 (10.3)	141.4 (8.3)	88.8
Cluster 2	-156.0 (8.1)	89.5 (8.7)	11.2
**GlcNAc β(1-4) GlcNAc**	**Phi**	**Psi**	**Pop(%)**
Cluster 1	-72.8 (8.9)	-106.6 (11.0)	96.7
Cluster 2	-82.1 (7.1)	-154.8 (6.8)	3.3
**Man β(1-4) GlcNAc**	**Phi**	**Psi**	**Pop(%)**
Cluster 1	-75.5 (11.0)	-123.5 (12.6)	94.6
Cluster 2	-62.42 (7.9)	72.2 (9.5)	4.4
**Man α(1-6) Man**	**Phi**	**Psi**	**Pop(%)**
Cluster 1	73.4 (10.5)	102.8 (10.6)	55.3
Cluster 2	70.4(9.4)	-176.8 (16.7)	26.7
Cluster 2	102.5 (9.3)	55.9 (7.7)	17.9
**GlcNAc β(1-2) Man (*1-6*)**	**Phi**	**Psi**	**Pop(%)**
Cluster 1	-91.8 (13.5)	159.4 (11.1)	100
**Gal β(1-4) GlcNAc (*1-6*)**	**Phi**	**Psi**	**Pop(%)**
Cluster 1	-74.2 (11.1)	-121.1 (15.3)	99.2
Cluster 2	-146.6 (7.5)	-143.6 (4.9)	0.8
**Man α(1-3) Man**	**Phi**	**Psi**	**Pop(%)**
Cluster 1	68.7 (9.7)	115.4 (16.8)	100
**GlcNAc β(1-2) Man (*1-3*)**	**Phi**	**Psi**	**Pop(%)**
Cluster 1	-79.5 (13.8)	162.3 (12.4)	92.4
Cluster 2	-79.3 (6.9)	110.3 (6.9)	7.6
**Gal β(1-4) GlcNAc (*1-3*)**	**Phi**	**Psi**	**Pop(%)**
Cluster 1	-72.6 (11.0)	-119.1 (15.2)	93.4
Cluster 1	-143.2 (10.4)	-144.4 (5.9)	4.9
Cluster 1	-74.2 (11.6)	69.7 (12.1)	1.7

Table S15. Results of the clustering analysis showing the median and standard deviation values (in parenthesis) for the torsion angles (°) measured through a cumulative 2 μs MD sampling of the β(1-2) xylosylated and α(1-3) fucosylated *A2* glycan terminating with LeX on both arms.

**Fuc α(1-3) GlcNAc**	**Phi**	**Psi**	**Pop(%)**
Cluster 1	-70.2 (11.0)	141.9 (8.4)	90.8
Cluster 2	-156.7 (7.6)	90.2 (8.6)	9.2
**GlcNAc β(1-4) GlcNAc**	**Phi**	**Psi**	**Pop(%)**
Cluster 1	-72.2 (8.6)	-107.5 (7.7)	100
**Man β(1-4) GlcNAc**	**Phi**	**Psi**	**Pop(%)**
Cluster 1	-76.4 (15.7)	-124.6 (16.7)	75.0
Cluster 2	-68.1 (9.7)	71.0 (10.1)	25.0
**Man α(1-6) Man**	**Phi**	**Psi**	**Pop(%)**
Cluster 1	70.7 (9.2)	-173.6 (13.4)	89.7
Cluster 2	152.4 (12.9)	145.6 (12.3)	10.3
**Xyl β(1-2) Man**	**Phi**	**Psi**	**Pop(%)**
Cluster 1	-77.6 (9.6)	140.1 (18.1)	100
**GlcNAc β(1-2) Man (*1-6*)**	**Phi**	**Psi**	**Pop(%)**
Cluster 1	-78.2 (14.3)	162.3 (13.1)	91.8
Cluster 2	-76.9 (7.4)	111.1 (6.8)	8.2
**Fuc β(1-3) GlcNAc (*1-6*)**	**Phi**	**Psi**	**Pop(%)**
Cluster 1	-69.7 (8.9	142.1 (7.0)	100
**Gal α(1-4) GlcNAc (*1-6*)**	**Phi**	**Psi**	**Pop(%)**
Cluster 1	-67.7 (8.0)	-107.9(7.2)	100
**Man α(1-3) Man**	**Phi**	**Psi**	**Pop(%)**
Cluster 1	61.5 (8.5)	111.0 (15.1)	100
**GlcNAc β(1-2) Man (*1-3*)**	**Phi**	**Psi**	**Pop(%)**
Cluster 1	-78.8 (13.9)	162.6 (11.7)	91.6
Cluster 2	-79.1 (6.2)	112.5 (6.2)	8.3
**Gal β(1-3) GlcNAc (*1-3*)**	**Phi**	**Psi**	**Pop(%)**
Cluster 1	-69.1 (9.45)	142.2 (8.0)	100
**Fuc α(1-4) GlcNAc (*1-3*)**	**Phi**	**Psi**	**Pop(%)**
Cluster 1	-67.8 (7.9)	-108.1 (7.4)	94.8
Cluster 2	-153.9 (8.8)	-141.5 (7.7)	5.2

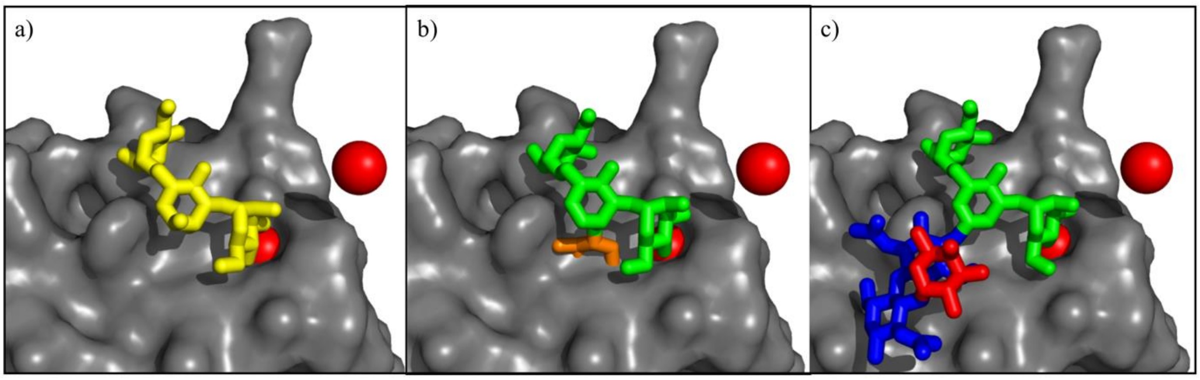

Figure S3. Structural alignment of different plant N-glycoforms from our MD simulations on to the DC-SIGN/GlcNAc2Man3 complex resolved at 2.5 Å resolution (PDBid 1k9i). Panel a) The water accessible surface of the DC-SIGN (chain C) binding site is shown in grey and the Man3 region of the co-crystallized glycan in 1k9i is rendered as yellow sticks. Ca^{2+} ions are shown as red spheres. Panel b) Structural alignment of representative structure from our MD simulation of the *ngx* plant N-glycan shows that the β(1-6) xylose sterically hinders binding by clashing with the surface of the binding site. Only the Xyl-Man3 glycoblock from the whole N-glycan is represented. Panel c) Structural alignment of representative structure from our MD simulation of the *ngf* plant N-glycan shows that the α(1-3) fucose does not hinders recognition or binding by DC-SIGN. Only the Man3 and α(1-3)-Fuc chitobiose glycoblocks from the whole N-glycan is represented. The monosaccharides colouring, aside from panel a), follows the SFNG nomenclature. The structure rendering was done with VMD and the graphical statistical analysis with *RStudio* (www.rstudio.com).

References

1. Case, D.; Ben-Shalom, I.; Brozell, S.; Cerutti, D.; Cheatham III, T.; Cruzeiro, V.; Darden, T.; Duke, R.; Ghoreishi, D.; Gilson, M.; Gohlke, H.; Goetz, A.; Greene, D.; Harris, R.; Homeyer, N.; Izadi, S.; Kovalenko, A.; Kurtzman, T.; Lee, T.; LeGrand, S.; Li, P.; Lin, C.; Liu, J.; Luchko, T.; Luo, R.; Mermelstein, D.; Merz, K.; Miao, Y.; Monard, G.; Nguyen, C.; Nguyen, H.; Omelyan, I.; Onufriev, A.; Pan, F.; Qi, R.; Roe, D.; Roitberg, A.; Sagui, C.; Schott-Verdugo, S.; Shen, J.; Simmerling, C.; Smith, J.; Salomon-Ferrer, R.; Swails, J.; Walker, R.; Wang, J.; Wei, H.; Wolf, R.; Wu, X.; Xiao, L.; York, D.; Kollman, P. *AMBER 2018*, University of California, San Francisco, 2018.
2. Kirschner, K. N.; Yongye, A. B.; Tschampel, S. M.; González-Outeiriño, J.; Daniels, C. R.; Foley, B. L.; Woods, R. J., GLYCAM06: a generalizable biomolecular force field. Carbohydrates. *J Comput Chem* **2008,** *29* (4), 622-55.
3. Jorgensen, W.; Chandrasekhar, J.; Madura, J.; Impey, R.; Klein, M., Comparison of simple potential functions for simulations of liquid water. *Journal of Chemical Physics* **1983,** *79* (2), 926-935.
4. Harbison, A. M.; Brosnan, L. P.; Fenlon, K.; Fadda, E., Sequence-to-structure dependence of isolated IgG Fc complex biantennary N-glycans: a molecular dynamics study. *Glycobiology* **2019,** *29* (1), 94-103.
5. Goga, N.; Rzepiela, A. J.; de Vries, A. H.; Marrink, S. J.; Berendsen, H. J., Efficient Algorithms for Langevin and DPD Dynamics. *J Chem Theory Comput* **2012,** *8* (10), 3637-49.
6. Allen, M.; Tildesley, D., *Computer simulation of liquids*. Clarendon Press: 1989.
7. Berendsen, H.; Postma, J.; van Gunsteren, W.; DiNola, A.; Haak, J., Molecular dynamics with coupling to an external bath. *J. Chem. Phys.* **1984,** *81*, 3684.
8. Ryckaert, J.-P.; Ciccotti, G.; HJC, B., Numerical Integration of the Cartesian Equations of Motion of a System with Constraints: Molecular Dynamics of *n* -Alkanes. *J Comp Phys* **1977,** *23* (3), 327-341.
9. Humphrey, W.; Dalke, A.; Schulten, K., VMD: visual molecular dynamics. *J Mol Graph* **1996,** *14* (1), 33-8, 27-8.
